# A survey on outcomes of accidental atovaquone–proguanil exposure in pregnancy

**DOI:** 10.1186/s12936-018-2352-z

**Published:** 2018-05-15

**Authors:** Kathrine R. Tan, Jessica K. Fairley, Mengxi Wang, Julie R. Gutman

**Affiliations:** 1 Malaria Branch, Center for Global Health, Centers for Disease Control and Prevention, 1600 Clifton Rd. MS A6, Atlanta, GA 30329 USA; 2 Department of Medicine, Division of Infectious Diseases, Emory University School of Medicine, 201 Dowman Dr., Atlanta, GA 30322 USA; 3 Department of Epidemiology, Rollins School of Public Health, Emory University, 1518 Clifton Rd, Atlanta, GA 30322 USA

**Keywords:** Malaria, Pregnancy, Prophylaxis, Atovaquone–proguanil

## Abstract

**Background:**

Malaria chemoprophylaxis options in pregnancy are limited, and atovaquone–proguanil (AP) is not recommended because of insufficient safety evidence. An anonymous, internet-based survey was disseminated to describe outcomes of pregnancies accidentally exposed to AP. Outcomes of interest included miscarriage (defined as pregnancy loss before 20 weeks), stillbirth (defined as pregnancy loss at or after 20 weeks), preterm birth or live birth prior to 37 weeks, and the presence of congenital anomalies.

**Results:**

A total of 487 women responded and reported on 822 pregnancies. Of the 807 pregnancies with information available on exposure and outcomes, 10 (1.2%) had atovaquone–proguanil exposure, all in the first trimester, and all resulted in term births with no birth defects.

**Conclusions:**

Use of an anti-malarial not recommended in pregnancy is likely to occur before the woman knows of her pregnancy. This study adds to the limited evidence of the safety of AP in pregnancy. Further study on use of AP in pregnancy should be a high priority, as an alternative option for the prevention of malaria in pregnancy in non-immune travellers is urgently needed.

## Background

Malaria infection during pregnancy is associated with increased risk of complications for both mother and fetus [1]. Pregnant women are advised to avoid or delay travel to malaria-endemic regions [2]. If avoiding travel is not feasible, pregnant women should take measures to avoid mosquito bites and also use an effective chemoprophylaxis regimen [2].

Among pregnant international travellers, malaria prevention is a frequent health concern. One study of travellers presenting to a traveller’s health clinic network found that while pregnant women comprised only a fraction of patients (0.8%), almost all pregnant travellers (95%) were traveling to malaria-endemic areas [3]. However, chemoprophylaxis options for pregnant women are very limited. Currently, only chloroquine and mefloquine are recommended for prophylactic use during pregnancy. Due to significant chloroquine resistance among *Plasmodium falciparum* parasites, use of chloroquine prophylaxis is restricted to just a few geographic areas, thus in most cases, mefloquine remains the only available option [2]. In some parts of South-East Asia, *P. falciparum* is also resistant to mefloquine, leaving pregnant women with no prophylaxis alternative [2]. Doxycycline, which is recommended in non-pregnant travellers, is contraindicated in pregnancy due to teratogenicity observed in animal fetuses, with detrimental effects on fetal teeth and bones [2]. Primaquine is also contraindicated in pregnancy, due to the possibility of haemolytic anaemia if the fetus has glucose-6-phosphate dehydrogenase deficiency [2].

Atovaquone–proguanil (AP, Malarone^®^) is a drug combination that is effective for malaria prophylaxis and treatment, even in regions with high rates of resistance to other anti-malarials [4]. Despite its efficacy, AP is not currently recommended for use by pregnant women due to insufficient data on its safety in pregnancy [5].

The individual drugs comprising AP also have limited information available for their use in pregnancy. Proguanil alone has a history of use for prevention of malaria, but there are no adequate controlled studies of its use in pregnant women [6]. Atovaquone has been used in pregnant women for the treatment of both toxoplasmosis and babesiosis, but does not have approval for use in pregnant women for either of these indications [7, 8].

In animal studies, there has not been evidence of teratogenic or embryotoxic effects at concentrations of atovaquone and proguanil corresponding to the estimated human exposure during treatment of malaria [9]. Adverse fetal effects, which consisted of decreased fetal body lengths as well as increased early resorptions and post-implantation losses, were observed in rabbits exposed to atovaquone alone at 1.3 times the estimated human exposure, resulting in maternal toxicity [9].

The limited data available from human studies of AP in pregnancy are studies of malaria treatment, and have not demonstrated an increased risk of adverse birth outcomes. A prospective study carried out in an area of Thailand with high rates of resistant malaria enrolled 81 pregnant women with uncomplicated malaria in their second or third trimesters of pregnancy. Women received either quinine sulfate or artesunate and AP orally. There were no differences in the birth weight or congenital abnormality rates in the infants between the two groups [10]. Another study in Thailand and Zambia demonstrated no serious adverse effects, including no stillbirths, spontaneous abortions, or birth defects among 26 women treated with AP in their third trimester for acute uncomplicated *P. falciparum* malaria [11].

AP has the potential to be a viable option for malaria prevention in pregnant travellers, but more evidence is needed. As AP is not recommended for pregnancy in the United States (US) or other countries, a study with intentional exposure to the drug is not feasible. It is possible, however, for accidental exposure to occur, especially in early pregnancy when a woman might still be unaware of her pregnancy, and takes AP as chemoprophylaxis. The objective of this study was to describe adverse pregnancy and birth outcomes among women who took AP during pregnancy, and to compare these outcomes to women who took chloroquine, mefloquine, or no anti-malarials at all.

## Methods

An anonymous, internet-based survey was conducted July 18–August 31, 2017. An invitation to participate was posted online to a national social media group of female physicians, and was e-mailed to all members of a listserv consisting of staff at the Centers for Disease Control and Prevention (CDC) and at Emory University. Weekly reminders were posted to maximize participation. Women who had at least one singleton pregnancy with an available birth or pregnancy outcome were included in the analysis. The survey included questions about all pregnancies, use of medication during pregnancy, type of medication including anti-malarials, whether the anti-malarial was taken for prevention or treatment of malaria, trimester during which exposure to the anti-malarial occurred (when applicable), and outcome of the pregnancy. Outcomes of interest included miscarriage (defined as pregnancy loss before 20 weeks), stillbirth (defined as pregnancy loss at or after 20 weeks), preterm birth or live birth prior to 37 weeks, and the presence of congenital anomalies.

Simple frequencies were used to describe pregnancies in terms of type of anti-malarial exposure, trimester of exposure based on self-report, and outcomes. Outcomes were further stratified by exposure to AP, chloroquine, mefloquine, or no anti-malarials during pregnancy. Because of the small numbers of pregnancies exposed to anti-malarials, no measures of association or tests of significance were calculated. Data were analysed using SAS v9.3 (SAS Institute, Cary, NC).

This study was determined to not require review by the Emory University Institutional Review Board, and was approved by CDC’s Institutional Review Board.

## Results

A total of 498 women responded, 487 of whom met eligibility requirements. These women reported 822 singleton pregnancies. Median age of women during pregnancy was 33 years (range 18–46 years). Of the 807 (98%) pregnancies with information on whether or not there was exposure to medications, 572 (70.9%) had exposure to any type of medication, and of these, 568 were able to recall if the medication was an anti-malarial. Of these 568, 32 (5.6%) were exposed to anti-malarials taken for malaria prophylaxis. Most (28/32, 87.5%) of the pregnancies were exposed to only one anti-malarial: 8 (25.0%) were exposed to AP, 14 (43.8%) to mefloquine, 5 (15.6%) to chloroquine or hydroxychloroquine, and 1 (3.1%) to an unknown anti-malarial. The four pregnancies with exposure to more than one anti-malarial were exposed to: AP, chloroquine and mefloquine; AP and doxycycline; chloroquine and mefloquine; and chloroquine and artemether–lumefantrine.

Outcomes were available for 32 pregnancies exposed to anti-malarials and 760 pregnancies unexposed to anti-malarials (Table 1). All ten pregnancies with exposure to AP and with a known outcome resulted in live term infants with no birth defects. The 21 pregnancies exposed to chloroquine, hydroxychloroquine, or mefloquine (excluding those with concurrent exposure to AP) resulted in 14 (71.4%) term births, 3 (9.5%) preterm deliveries, 2 (9.5%) miscarriages, and 2 (9.5%) stillbirths. Two birth defects, an incomplete syndactyly and a right branchial cleft anomaly, were reported among pregnancies exposed to anti-malarials, both in term infants exposed to mefloquine in utero in the first and third trimesters, respectively. Both were minor anomalies not requiring medical intervention. Among the 760 pregnancies with no anti-malarial exposure, including pregnancies with no medication exposure at all, 632 (83.2%) were term deliveries, 50 (6.6%) were preterm deliveries, 69 (9.1%) were miscarriages, 3 (0.4%) were stillbirths, and 6 (0.8%) were electively terminated. Among pregnancies with no exposure to anti-malarials, the overall rate of birth defects was 3.8% (29/760), and the defects were varied with the most common being cleft lip/palate 13.8% (4/29); 65.5% (19/29) of infants with birth defects required surgical intervention.

**Table 1:** Description of outcomes of pregnancies by exposure

	Live term	Live preterm	Miscarriage	Stillbirth	Termination
Atovaquone–proguanil (n = 10)	10^a^	0	0	0	0
Chloroquine (n = 6)	1	3^b^	1	1	0
Mefloquine (n = 15)	13^c,d^	0	1	1	0
Unknown anti-malarial (n = 2)	0	1	1	0	0
No anti-malarials (n = 760)	632^e^	50	69	3	6
No drugs at all (n = 235)^f^	181	13	32	1	3

^a^Includes two pregnancies with additional anti-malarial exposures in addition to atovaquone–proguanil: one to doxycycline, another to both chloroquine and mefloquine

^b^Includes one pregnancy also exposed to artemether–lumefantrine and one pregnancy exposed to plaquenil (considered equivalent to CQ)

^c^Includes one pregnancy with chloroquine exposure

^d^Birth defects reported in 2 infants: incomplete syndactyly and right branchial cleft anomaly, no surgical intervention needed for either

^e^Overall rate of birth defects was 3.8% (29/760), and the defects were varied with the most common being cleft lip/palate 13.8% (4/29); 65.5% (19/29) of infants with birth defects required surgical intervention

^f^Five pregnancies had unknown outcome. Overall rate of birth defects was 1.4% (4/280): congenital hydronephrosis, ambiguous genitalia, and 2 unspecified

## Conclusions

Among respondents to this survey, use of any anti-malarial during pregnancy, primarily chloroquine or mefloquine, which are indicated in pregnancy, was not uncommon. Furthermore, use of an anti-malarial not recommended in pregnancy is likely to occur before the woman knows of her pregnancy, as demonstrated by the first trimester exposure to AP in all 10 pregnancies with any AP exposure, and in one case, doxycycline. All reported pregnancies with any AP exposure resulted in live term births with no birth defects.

This study has several limitations. The number of invited individuals is unknown because it is not known how many individuals saw the posted invitation on social media, and it is unknown what proportion of the listserv is male, in which case, the invitation would not apply. Also, the sample size of this survey was too small for comparisons of outcomes between pregnancies exposed to AP compared to other anti-malarials, and depended on self-report for exposure and outcomes.

These findings add to the limited evidence of the safety of AP in pregnancy. Previously mentioned animal studies [9] suggest the biologic plausibility of the safety of AP, and the aforementioned studies in Zambia and Thailand in which AP was used for treatment of malaria with no adverse outcomes [10, 11] suggest that AP is safe in the third trimester. Another study, a Danish registry-based study of a cohort of 570,877 live births investigated AP exposure in early pregnancy. There were 149 women exposed to AP during pregnancy, and the study found no significant association between exposure to AP between 3 and 8 weeks after conception (n = 134) and any major birth defects [12].

While the available data on the safety of AP in pregnancy might not be sufficient at this time to support interventional studies on AP for prevention of malaria in pregnant travelers from non-endemic countries, cohort studies using existing data sources, such as registry-based studies or medical claims data to examine safety of AP in pregnancy may provide additional evidence to justify interventional studies on prevention or treatment of malaria in pregnant women in endemic areas where the benefits may outweigh the risks. Further study on use of AP in pregnancy should be a high priority, as an alternative option for the prevention of malaria in pregnancy in non-immune travellers is urgently needed.
